# Dung‐associated arthropods influence foraging ecology and habitat selection in Black‐necked Cranes (*Grus nigricollis*) on the Qinghai–Tibet Plateau

**DOI:** 10.1002/ece3.4904

**Published:** 2019-01-28

**Authors:** Wei Liu, Yongjie Wu, Shane G. DuBay, Chenhao Zhao, Bin Wang, Jianghong Ran

**Affiliations:** ^1^ Key Laboratory of Bio‐resources and Eco‐environment of Ministry of Education, College of Life Sciences Sichuan University Chengdu China; ^2^ College of Life Sciences Huaibei Normal University Huaibei China; ^3^ Committee on Evolutionary Biology University of Chicago Chicago Illinois; ^4^ Life Sciences Section, Field Museum of Natural History Integrative Research Center Chicago Illinois

**Keywords:** birds, foraging availability, grassland, grazing activity, invertebrate prey accessibility

## Abstract

Variation in grassland vegetation structure influences the habitat selection of insectivorous birds. This variation presents a trade‐off for insectivorous predators: Arthropod abundance increases with vegetation height and heterogeneity, but access to arthropod prey items decreases. In contrast, grazing by large herbivores reduces and homogenizes vegetation, decreasing total arthropod abundance and diversity. However, the presence of livestock dung may help counteract the overall reduction in invertebrates by increasing arthropods associated with dung. It is unclear, however, how the presence of arthropod prey in dung contributes to overall habitat selection for insectivorous birds or how dung‐associated arthropods affect trade‐offs between vegetation structure, arthropod abundance, and access to prey. To explore these relationships, we studied habitat selection of the Black‐necked Crane (*Grus nigricollis*), a large omnivorous bird that breeds on the Qinghai–Tibet Plateau. We assessed the relationships between habitat selection of cranes and vegetation structure, arthropod abundance, and the presence of yak dung. We found that Black‐necked Cranes disproportionately foraged in grassland patches with short sward height, low sward height heterogeneity, and high numbers of dry yak dung, despite these habitats having lower total arthropod abundance. Although total arthropod abundance is lower, these habitats are supplemented with dry yak dung, which are associated with coleopteran larvae, making dung pats an indicator of food resources for breeding Black‐necked Cranes. Coleopteran adults and larvae in yak dung appear to be an important factor influencing the habitat selection of Black‐necked Cranes and should be considered when assessing grassland foraging trade‐offs of insectivorous birds. This research provides new insights into the role of livestock dung in defining foraging habitats and resources for insectivorous predators.

## 1 INTRODUCTION

In grassland ecosystems, variation in vegetation structure poses a trade‐off for insectivorous birds between abundance and access to arthropod prey: The abundance and diversity of arthropods increase with vegetation height and heterogeneity (Dennis et al., 2008; Kruess & Tscharntke, 2002), but at the same time access to these prey items decreases (Butler & Gillings, 2004; Devereux, McKeever, Benton, & Whittingham, 2004). Habitat selection of insectivorous birds is thought to balance this vegetation structure trade‐off. Previous work suggests that insectivorous grassland birds select foraging habitats with low vegetation height but high vegetation height heterogeneity (e.g., Atkinson et al., 2005; Devereux et al., 2004; Evans et al., 2006; Vandenberghe, Prior, Littlewood, Brooker, & Pakeman, 2009), which may represent a compromise between prey abundance and prey accessibility (Benton, Vickery, & Wilson, 2003; Fuller & Gough, 1999; Perkins et al., 2000).

Given that vegetation structure in grassland ecosystems is altered through grazing activity from native herbivores and domesticated livestock (van Klink et al., 2013), the presence of herbivores has consequences on arthropods in these systems. As grazing pressure increases, sward height and heterogeneity decrease, which impacts arthropod abundance and diversity in the system (Atkinson et al., 2005; Zhu, Wang, Guo, Liu, & Wang, 2015). However, at the same time, small and large herbivores deposit dung throughout the grazed landscape, which may locally increase arthropod abundance around dung pats (Benton, Bryant, Cole, & Crick, 2002; Howe, Zorn‐Arnold, Sullivan, & Brown, 2006; McCracken & Foster, 1994; Vandegehuchte, Raschein, Schütz, Gwiazdowicz, & Risch, 2015). Previous work has suggested that dung‐associated arthropods may be an important invertebrate resource for insectivorous birds (Atkinson, Buckingham, & Morris, 2004), but the extent to which dung‐associated arthropods contribute to overall habitat selection in insectivorous birds is unclear. Here, we test how domesticated livestock impact habitat selection in a large omnivorous bird, the Black‐necked Crane (*Grus nigricollis*), by assessing the relationships between foraging habitat selection, vegetation structure, livestock dung, and arthropod abundance within the dung itself. By explicitly accounting for livestock dung, we can assess the role of dung in vegetation structure trade‐offs (i.e., abundance of vs. access to arthropod prey items) and test whether dung‐associated arthropods might mitigate the impacts of reduced arthropod prey as vegetation height and heterogeneity decrease with increases in grazing.

The Black‐necked Crane is a large omnivorous bird that breeds on the Qinghai–Tibet Plateau of central Asia, one of the world's most extensively grazed grassland systems (Klein, Harte, & Zhao, 2007). Like other omnivorous species that occupy grasslands elsewhere (e.g., Red‐crowned crane [*Grus japonensis*] and Great Bustard [*Otis tarda*]), the Black‐necked Crane shifts its diet toward animal foods during the breeding season, relying heavily on a diet of invertebrate prey to successfully rear offspring (Bravo, Ponce, Palacín, & Carlos Alonso, 2012; Hashimoto & Jin, 1981; Zhang & Ma, 2011; Zhao, Wan, Wang, & Gao, 2007). The Qinghai–Tibet Plateau has a long history of large grazing mammals (Schaller, 1998), such as wild yak (*Bos mutus*) and Tibetan gazelle (*Procapra picticaudata*), which impact vegetation structure and arthropod communities. Over the past millennia, native herbivores have been largely replaced by domestic yak (*Bos grunniens*) and sheep (*Ovis aries*) (Miller, 1998). The intensity of grazing from domesticated livestock varies spatially and temporally across the Qinghai–Tibet Plateau, creating a dynamic and heterogeneous grassland landscape. These gradients of grazing pressure provide a natural experimental setting to explore the relationships among vegetation structure, arthropod abundance, livestock dung, and habitat selection of Black‐necked Cranes. In this study, we quantified foraging habitat selection and diet of Black‐necked Cranes in relation to vegetation structure and arthropod abundance and diversity on the grassland surface and within domestic yak dung.

## 2 MATERIALS AND METHODS

### 2.1 Study system

**Figure 1:**
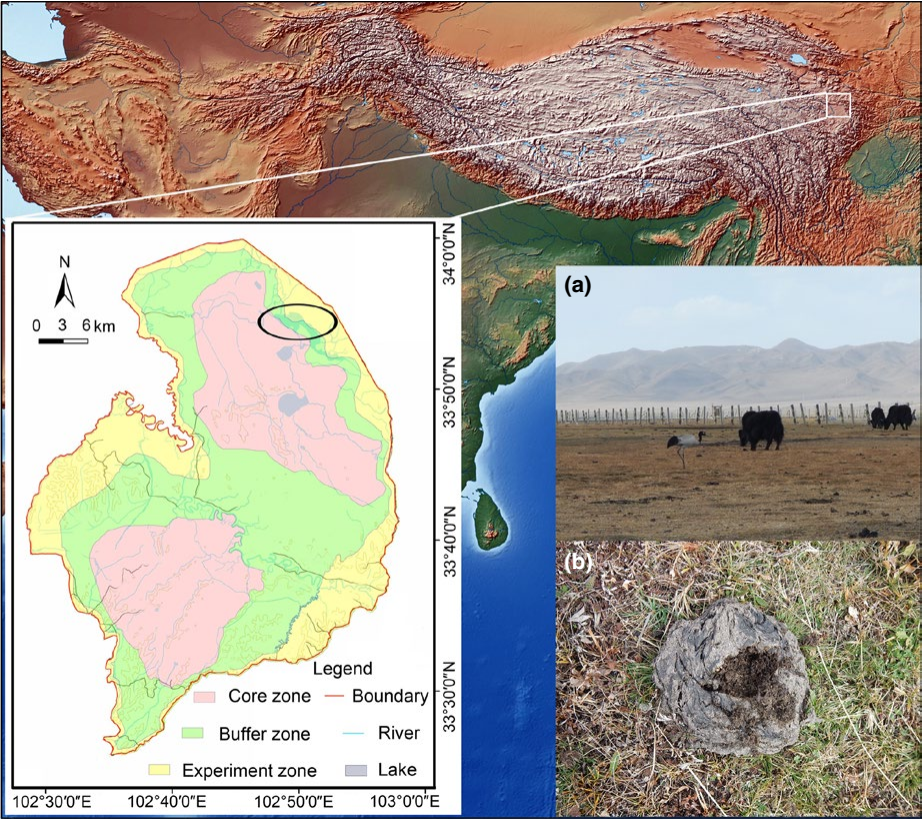
Location of study area. The ellipse on the bottom left map shows pasture patches used for observation in the Zoige National Wetland Natural Reserve. Photographs of foraging habitat of Black‐necked Crane on the bottom right shows (a) foraging Black‐necked Crane in meadow habitat with yaks; (b) dry yak dung opened by Black‐necked Crane

We conducted this study at the Zoige Wetland (elevation 3,400–3,500 m) on the eastern Qinghai–Tibet Plateau (33.89°N–33.94°N, 102.795°E–102.887°E, ~33 km^2^, Figure 1) over two consecutive breeding seasons (May–September in 2015 and 2016). The Zoige Wetland is one of the five most important grazing regions in China. This high‐elevation grassland system harbors a number of endemic and endangered species (Myers, Mittermeier, Mittermeier, Fonseca, & Kent, 2000; Scott, 1993), including the Black‐necked Crane, which is the only extant crane that breeds on the Qinghai–Tibet Plateau (Figure 1). The Black‐necked Crane is currently listed as “Vulnerable” on the IUCN Red List (BirdLifeInternational, 2016). The Zoige Wetland Nature Reserve protects 1,666 km^2 ^of habitat and was established in 1998 to conserve the breeding habitats of the Black‐necked Crane and other waterbirds, as well as protect a vulnerable high plateau wetland ecosystem (Wu, Zha, Zhang, & Yang, 2009). The Zoige Wetland Nature Reserve is the largest breeding and summering area for Black‐necked Cranes (Ran et al., 1999; Scott, 1993), and habitats in this wetland are classified as follows: marsh, marsh meadow, and meadow (Han, Yang, Yang, & Li, 2011; Tian, 2005).

### 2.2 Characterizing foraging habitats of the Black‐necked Crane

Black‐necked Cranes have fixed territories (about 1–3 km^2^ per pair) during the breeding season (Dwyer, Bishop, Harkness, & Zhong, 1992; Li & Li, 2005). In 2015, we observed 13 breeding pairs from March to September at Huahu Marsh, the area within the Zoige wetlands with the highest density of cranes (Dou et al., 2013). Breeding home ranges covered marsh, marsh meadow, and meadow habitat. Foraging behavior of each pair was observed throughout four breeding stages, except when pairs did not lay eggs (*n* = 3) or the offspring died after hatching (*n* = 3). The four breeding stages were as follows: pre‐incubation (before the egg‐laying date, March–June 2015), incubation (from onset of egg laying to the date of hatching, April–July 2015), post‐fledging (from the date of hatching to when nestling cranes start to fly, May–September 2015), and full‐fledged (from when nestling cranes flew until they departed on migration, July–October 2015). During each observation period, foraging behavior and prey capture were observed using a spotting scope for 2 hr from underneath a camouflage coat, at a minimum distance of 100–150 m from the cranes (Buchanan et al., 2012). All observations were conducted between the hours of 08:00 and 17:00 (Jiang, 2015). For the pre‐incubation stage, each pair was observed 2–4 times (because the duration of this stage varied), while other stages were observed four times.

All observed foraging locations for the 13 breeding pairs were drawn on a detailed map (1:50,000). The most frequently visited foraging location during each observation period was defined as a 200 m^2^ foraging square quadrat (Supporting Information Figure S1b, hereafter referred to as the foraging squares). For each foraging square, we determined a complementary random square to compare the vegetation structure in the cranes’ foraging habitat (i.e., the foraging squares) to the general grassland environment (i.e., the random squares). We established random squares by throwing a wooden stick straight up and allowing it to fall to the ground (van Klink, Mandema, Bakker, & Tinbergen., 2014). We used the direction of the stick to establish the position of each random square, which was 100–150 m away from the foraging square to ensure that vegetation composition was generally similar between the foraging and random squares. The direction and distance of each random square were determined from the center of its paired foraging square (Buchanan et al., 2012). We set up a paired foraging square and random square for each foraging observation.

In each foraging square and random square (*n* = 290, Supporting Information Figure S1b), we (a) measured sward height and sward height heterogeneity, (b) conducted a vegetation survey, and (c) counted the number of yak‐dung pats of varying wetness categories in 15 (1 m^2^) plots. For the vegetation height measurements, we used a direct measurement method (Hodgson, Tayler, & Lonsdale, 1971; Stewart, Bourn, & Thomas, 2001), binning data into three height classes: 0–10 cm, 10–20 cm, and >20 cm (Durant, Tichit, Fritz, & Kerneis, 2008). We calculated height heterogeneity as *H* = *∑*[*P*(*i*, j) × log_10_ *P*(*i*, *j*)], where *i* and *j* are two height classes and *P*(*i*, *j*) is the probability of finding these two classes adjacent along the transect (the transect method design can be found in Supporting Information Figure S1b; Burel et al., 1998). Mean sward height and sward height heterogeneity were normalized using log_10_ or log_10_ (*X* + 1) before analyses. The three wetness categories for yak dung were defined as follows: fresh (<12 hr old), sub‐dry (the surface of dung was dry but inside was wet), and dry (the entire dung was dry).

Binomial‐distributed generalized linear mixed models (GLMMs) were used to assess the differences in mean sward height, sward height heterogeneity, and the number of yak dung between the foraging squares and random squares. The average values for sward height in the 15 plots (Supporting Information Figure S1b) and sward height heterogeneity per square were used in the analyses with a nested design structure (pair/breeding stage) specified as a random effect. The difference in the number of fresh, sub‐dry, and dry yak‐dung pats between both foraging square and random square was tested using GLMM with the same design structure.

### 2.3 Diet composition of the Black‐necked Crane

To study the diet of Black‐necked Cranes in their foraging habitats, we sampled the undigested invertebrate remains found in crane feces. We collected 72 fresh fecal samples from the 13 breeding crane pairs between March and October in 2015. The Black‐necked Crane and the Greylag Goose (*Anser anser*) are the only large waterfowl in the study areas, and their feces are easily distinguishable from one another by visual inspection because the Greylag Goose is strictly herbivorous while the Black‐necked Crane is omnivorous. In the field, we placed each crane fecal sample into a 50‐ml tube containing 70% ethanol for later dissection and identification of invertebrate remains (Moreby, 1988; van Klink et al., 2014). For each pair of cranes, 2–11 samples (5.5 on average) were collected. In the laboratory, all invertebrate parts were identified to their taxonomic order: Araneae, Coleoptera, Hymenoptera, Diptera, Lepidoptera, and Orthoptera (Moreby, 1988; Ralph, Stephanie, & Ralph, 1985; van Klink et al., 2014). It should be noted that the sorting of prey items does not reflect the whole diet of the crane, as soft‐bodied arthropods, like worms, may be fully digested and difficult to identify, but the presence of invertebrates with exoskeletons can be easily detected (Moreby & Stoate, 2000). Soft‐bodied prey items with some hard parts, such as the mandibles of caterpillars, were identifiable in the crane fecal samples. For every sample, we determined the minimum number of prey items of each group by matching the parts found (van Klink et al., 2014). For example, we assumed that unidentifiable parts belonged to an individual for which other parts were identified, and because individual invertebrates possess two or more of many parts (e.g., jaws, legs, or wings), if matching left and right parts were present, we assumed these parts belonged to the same individual. Therefore, the numbers counted can be treated as the minimum number of individual invertebrates eaten by a Black‐necked Crane.

We also assessed changes in Black‐necked Crane diet over the four breeding stages. We used Poisson‐distributed GLMMs, because the number of individuals is countable, to test differences in the number of individuals between invertebrate groups in fecal samples. We specified a nested design structure (nest/breeding stage/fecal sample) as a random effect, prey number as a dependent variable, and prey group as an explanatory variable. The differences in the occurrence frequency of the different invertebrate groups over the samples were assessed with binomial GLMMs.

### 2.4 The relationship between invertebrate prey abundance, vegetation structure, and the prevalence of yak dung

To test the general relationships between vegetation structure, invertebrate prey abundance, and the number of yak dung on the grassland surface, we established 12 additional sampling plots (0.03 km^2^ in size) in 2015 across three sampling areas that differ in grazing intensity in Huahu Marsh (each sampling area contained four replicate plots). The three sampling areas were all within crane territories, and the sampling plots were established randomly without reference to the position of crane nests. The first sampling area was in winter pasture, where grazing is banned from May to October each year (beginning in 2010, which we determined from interviewing members from the local community). The second sampling area was in rotational grazing pasture, where grazing is banned in May, July, and September (each grazing month is about 30 days [8.5 hr per day]). The third sampling area was in resettled habitat for tents and livestock where grazing is the highest and uninhibited. All three sampling areas were established outside areas where local herdsmen collect yak dung for fuel, as the yak dung from stable areas is generally sufficient to meet fuel needs. Each of the 12 replicate plots had ten 200‐m^2 ^quadrats that we surveyed (the diagram of the ten quadrats is shown in Supporting Information Figure S1a). We surveyed each of the 120 sampling quadrats in May, July, and September in 2015, for a total of 360 surveys (three sampling areas × 4 plots × 10 quadrats × 3 times). The quadrats thus accounted for 20% of the three sample areas (the difference in vegetation and invertebrates among three sampling areas is found in Supporting Information Table S1).

In each of the 120 sampling quadrats, we (a) measured vegetation height by a direct measurement method (Hodgson et al., 1971; Stewart et al., 2001), (b) sampled aboveground invertebrates, and (c) counted the number of yak dung. To measure vegetation height, we placed a hand lightly on the vegetation at the level below which approximately 80% of the vegetation is estimated by eye to be growing (thus ignoring occasional tall stalks). We then took a reading of this height with a ruler (Hodgson et al., 1971; Stewart et al., 2001). Surface‐active invertebrates were sampled using pitfall traps, which were set for 1 week (Atkinson et al., 2005; Li, 2006); Supporting Information Figure S1b). In each quadrat, five pitfall traps were set (as detailed in Supporting Information Figure S1b) from March to September 2015 (overlapping with the period in which we investigated the diet of the crane). We counted and sorted all invertebrates larger than 5 mm in length belonging to the six orders Araneae, Coleoptera, Hymenoptera, Diptera, Lepidoptera, and Orthoptera (van Klink et al., 2014) and stored them in 70% ethanol. Adults and larva were captured by pitfall traps. Total invertebrate abundance was calculated as the sum of all individuals of the six orders per square.

Between June and August in 2016, we sampled a total of 199 yak‐dung pats in rotational grazing pasture. We chose this type of pasture because we could find fresh, sub‐dry, and dry yak‐dung pats simultaneously. Each pat was placed in one of the three moisture categories described above (Jiang & Zhou, 2005; McCracken & Foster, 1994), and only unbroken dung pats were sampled. Invertebrates from each dung pat were separated by hand, and invertebrates were classified into the five orders: Araneae, Coleoptera, Hymenoptera, Diptera, and Dermaptera. Only individuals with body length >5 mm were identified, and all invertebrates were subsequently released to their original position in the square.

We used ordinal logistic‐generalized linear models (GLM) to test differences in the abundance of Coleopteran larvae, Coleopteran adults, Diptera larva, and other invertebrate prey items between the three categories of yak‐dung pats (fresh, sub‐dry, and dry; Bates, Maechler, & Bolker, 2013). We used the three wetness categories as the dependent variable, and the numbers of each invertebrate category (i.e., Coleopteran larvae) as the explanatory variable.

To compare the diet preference of the Black‐necked Crane to surface‐active invertebrate prey collected by pitfall traps, we used Mann–Whitney *U* tests to test difference in relative abundance of invertebrate groups (Coleopteran adults, Coleopteran larvae, Hymenoptera, Diptera, Lepidoptera, and Araneae) present between the crane fecal samples (*n* = 72) and the field samples (*n* = 360). To emphasize the putative importance of yak dung for cranes, we used Mann–Whitney *U* tests to test the difference in relative abundance of invertebrate groups (Coleopteran adults, Coleopteran larvae, and Diptera) between pitfall traps (*n* = 360) and the three categories of yak‐dung pats (fresh, *n* = 64; sub‐dry, *n* = 73; and dry, *n* = 62).

## 3 RESULTS

### 3.1 Foraging habitats of the Black‐necked Crane

**Figure 2:**
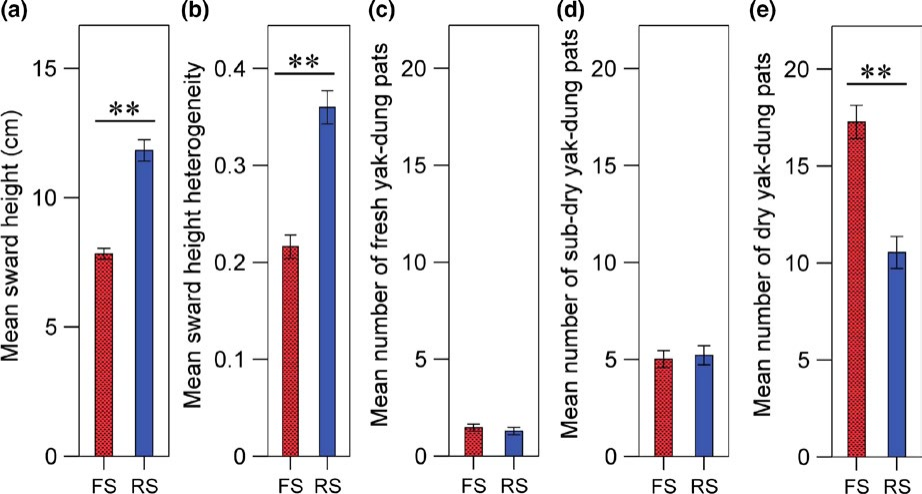
The differences of foraging and random squares in the following: (a) mean sward height, (b) sward height heterogeneity, (c) mean number of fresh pats, (d) mean number of sub‐dry pats, and (e) mean number of dry pats. Standard errors (±1 *SE*) are represented by vertical bars in (a–e). ^**^Denotes significant differences (*p* < 0.05). FS: foraging squares; RS: random squares

The number of cranes foraging in meadow habitat was higher than the proportion foraging in marsh and marsh meadow habitat (Supporting Information Table S2), and foraging occurred primarily in habitat with lower mean sward height and lower sward heterogeneity, which is reflected in the comparison of vegetation structure between foraging squares and random squares (Figure 2a,b). Mean sward height (GLMM, *F*_1,288_ = 75.15, *p* < 0.001, Figure 2a) and sward height heterogeneity (GLMM, *F*_1,288_ = 73.99, *p* < 0.001, Figure 2b) were significantly lower in foraging squares than in random squares (*n* = 290). Between the crane foraging squares and random squares, we found no significant differences in the number of fresh dung pats (GLMM, *F*_1,288_ = 1.56, *p* = 0.212) or sub‐dry dung pats (GLMM, *F*_1,288_ = 0.53, *p* = 0.468; Figure 2c,d). We did, however, find a significantly higher number of dry dung pats in foraging squares than in random squares (GLMM, *F*_1,288_ = 231.40, *p* < 0.001; Figure 2e).

### 3.2 Diet composition of the Black‐necked Crane

**Figure 3:**
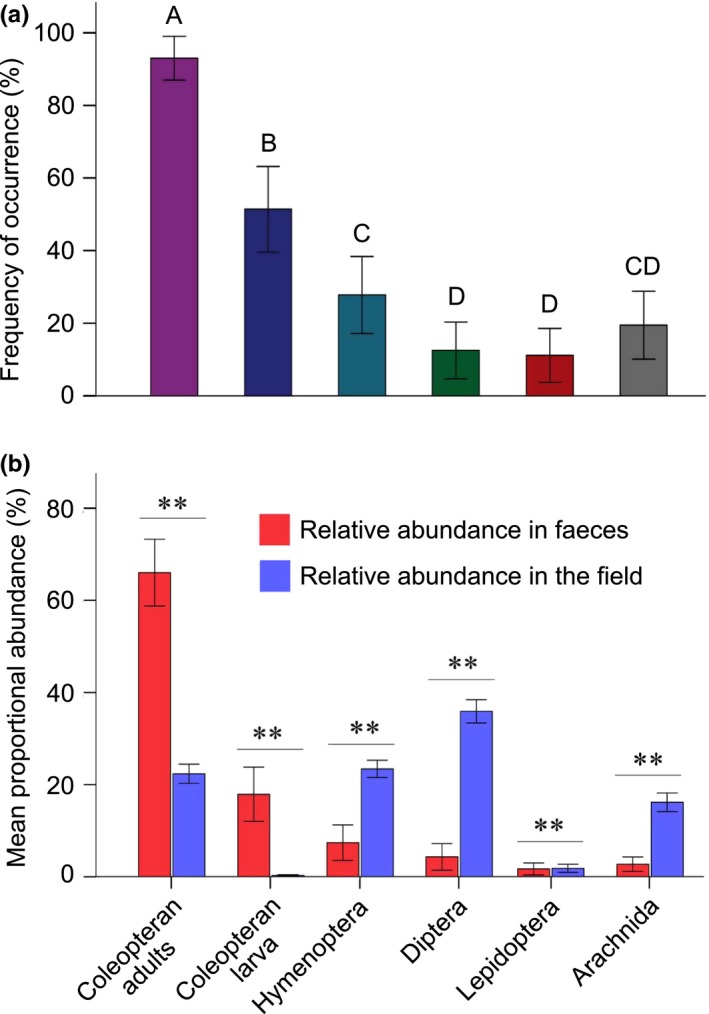
Diet composition of Black‐necked Crane based on 72 fecal samples of 13 breeding pairs from March to October in 2015 at Zoige Wetland, China. (a) Frequency of occurrence of the different invertebrate groups over all samples (% ± 95% CI). The letters above the bars denote pairwise statistical differences in which bars with different letters are statistically significant at *p* < 0.05, while bars with similar letters are not statistically different from one another (i.e., *p* > 0.05). The different colors represent the different prey items; (b) difference in relative abundance (% ± 95% CI) of all invertebrate groups presented in fecal samples (*n* = 72) and in field samples (*n* = 360), ^**^ *p* < 0.05 indicates the significant difference. Few Coleopteran larvae (not zero) were collected in the field.

From the 72 crane fecal samples, in only two fecal samples (2.8%) were arthropods not found. We identified 1,076 invertebrate individuals in the other 70 samples. Coleopteran adults comprised 60.1% of identified arthropod prey items, and Coleopteran larvae comprised 19.7%. Other prey groups in fecal samples were as follows: Hymenoptera (8.7%), Diptera (5.9%), Lepidoptera (2.8%), and Araneae (2.8%). Coleopteran adults were present in 93% of the samples, Coleopteran larvae 51%, Hymenoptera 28%, Diptera 13%, Lepidoptera 11%, and Araneae 19% (Figure 3a). Differences in occurrence between these arthropod groups were significant (GLMM: *F*_5,426_ = 200.04, *p* < 0.001), while Coleopteran adults were significantly more abundant than any other group.

### 3.3 The relationship between invertebrate prey abundance, vegetation structure, and yak dung

**Figure 4:**
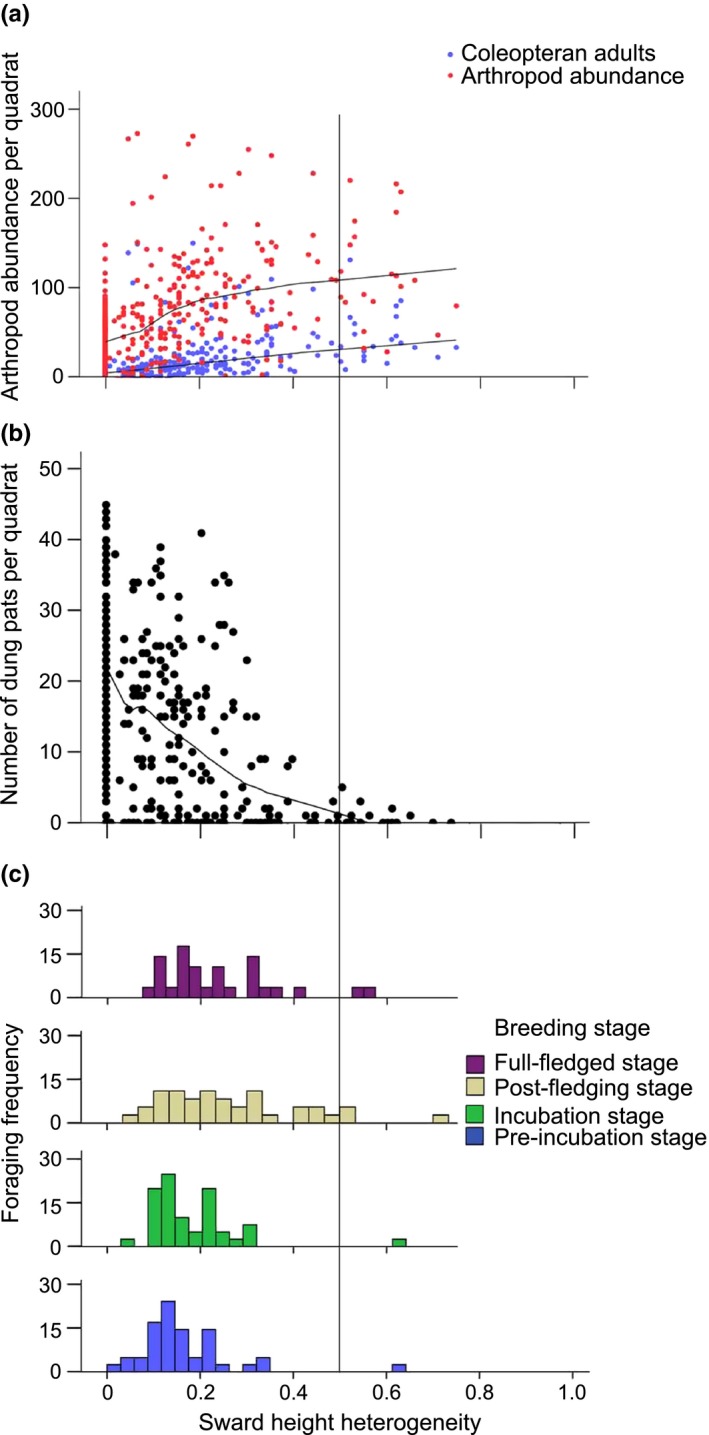
Relationships between mean sward height and (a) arthropod abundance (calculated as the number of individuals from five pitfall traps per sampling quadrat), (b) number of dung pats per sampling quadrat, and (c) crane habitat selection (calculated as the number of foraging events in a habitat with a given sward height), compiled from foraging square vegetation data. Foraging frequency is defined as the frequency that cranes foraged in a habitat of a given sward height during the four breeding stages. Fitted LOESS curves (50% of points fit) are shown for significant relationships in (a) and (b)

From the 120 quadrats (surveyed three times each) that were established across the three sampling areas of differing grazing intensity, a total of 25,610 arthropods were collected from pitfall traps. The relative abundance of each arthropod order can be found in Figure 3b. Mean sward height and heterogeneity were positively correlated with the total arthropod abundance and Coleopteran abundance (*R* ^2^ = 0.67, *p* < 0.001; *R* ^2^ = 0.75, *p* < 0.001, respectively, Figure 4a and Supporting Information Figure S3a). We also found a significant negative correlation between mean sward height and the number of dung pats (*R* ^2^ = 0.21, *p* < 0.001, Figure 4b), as expected by the established relationship between vegetation structure and invertebrate biomass. A correlation matrix for means sward height, total arthropod abundance, Coleopteran abundance, and the number of yak dung in the field is attached as an appendix (Supporting Information Table S3).

We investigated 199 yak‐dung pats and identified 3,178 invertebrate individuals in the dung. This analysis was focused on identifying Diptera, Coleoptera, and other orders (i.e., Dermaptera). For fresh dung pats (*n* = 64), Dipteran larvae made up 100% of invertebrate abundance. For sub‐dry dung pats (*n* = 73), Dipteran larvae made up 83.3% of invertebrate abundance, Coleopteran larvae made up 0.2%, Coleopteran adults made up 16.0%, and other entities (Dermaptera, Hymenoptera, and Araneae, etc.) made up 0.5%. For dry dung pats (*n* = 62), Dipteran larvae made up 0.7% of invertebrate abundance, Coleopteran larvae made up 96.3%, Coleopteran adults made up 1.7%, other entities (Dermaptera, Hymenoptera, and Araneae, etc.) made up 1.3% (Supporting Information Figure S2). There were significant differences in the abundance of Coleopteran adults, Coleopteran larvae, and Diptera larva between the three freshness categories of yak dung (GLM: Wald *X* ^2^ = 12.711, *p* < 0.001; Wald *X* ^2^ = 33.920, *p* < 0.001; Wald *X* ^2^ = 16.532, *p* < 0.001) but no significant difference in the abundance of other items (GLM: Wald *X* ^2^ = 0.828, *p* = 0.363; Table 1).

**Table 1:** Invertebrate abundance of different wetness categories of yak‐dung pats (mean ± *SE*)

Dung pats	Number of samples	Coleopteran adults	Coleopteran larva	Diptera larva	Other items
Fresh dung	64	0.0 ± 0.0	0.0 ± 0.0	0.3 ± 0.2	0.0 ± 0.0
Sub‐dry dung	73	5.4 ± 0.4	0.1 ± 0.0	28.0 ± 1.6	0.2 ± 0.1
Dry dung	62	0.2 ± 0.1	10.9 ± 0.7	0.1 ± 0.1	0.1 ± 0.1

### 3.4 Comparisons of arthropod abundance among Black‐necked Crane feces, the surface of the grassland, and yak dung

Comparison of arthropod abundance between crane fecal samples and pitfall traps suggests strong prey selection in Black‐necked Cranes (Figure 3b). Coleopteran adults and larva were disproportionately prevalent in crane feces given their relative low abundance on the surface of the grassland environment (Mann–Whitney *U* = 3,184.00, *p* < 0.001; Mann–Whitney *U* = 6,623.00, *p* < 0.001, Figure 3b). In contrast, Hymenoptera, Diptera, and Araneae were rare in fecal samples, but relatively abundant in pitfall traps (Mann–Whitney *U* = 5,103.00, *p* < 0.001; Mann–Whitney *U* = 2,777.00, *p* < 0.001; Mann–Whitney *U* = 5,732.00, *p* < 0.001, Figure 3b). Although Lepidoptera prevalence in pitfall traps was close to that of the relative abundance in fecal samples (Figure 3b), its relative abundance in the pitfall traps was significantly more than that of the fecal samples (Mann–Whitney *U* = 10,110.00, *p* = 0.003).

Coleopteran adults were relatively abundant in pitfall traps compared to Coleopteran adults in fresh yak‐dung pats (Mann–Whitney *U* = 87.50, *p = *0.001) and dry yak‐dung pats (Mann–Whitney *U = *1,740.50, *p < *0.001), but there was no significant difference between Coleopteran adult abundance in pitfall traps and sub‐dry yak‐dung pats (Mann–Whitney *U = *11,577.50, *p = *0.183). In contrast, Coleopteran larva was relatively rare in pitfall traps compared to Coleopteran larva in dry yak‐dung pats (Mann–Whitney *U = *0, *p < *0.001). There were no significant differences between Coleopteran larva abundance in pitfall traps and fresh yak‐dung pats (Mann–Whitney *U = *760.00, *p = *0.377) and sub‐dry yak‐dung pats (Mann–Whitney *U = *11,876.00, *p = *0.074). Dipterans were rare in pitfall traps but relatively abundant in fresh yak‐dung pats (Mann–Whitney *U = *7.50, *p < *0.001) and sub‐dry yak‐dung pats (Mann–Whitney *U = *1,950.50, *p < *0.001), but more abundant in pitfall traps than in dry yak‐dung pats (Mann–Whitney *U = *1,359.50, *p < *0.001).

Coleopteran adults were relatively abundant in fecal samples of Black‐necked Cranes, but rare in fresh yak‐dung pats (Mann–Whitney *U* = 7.50, *p < *0.001), sub‐dry yak‐dung pats (Mann–Whitney *U = *524.50, *p < *0.001), and dry yak‐dung pats (Mann–Whitney *U = *110.00, *p < *0.001). The abundance of Coleopteran larvae in crane fecal samples was more abundant than in fresh yak‐dung pats (Mann–Whitney *U = *82.50, *p* = 0.035) and sub‐dry yak‐dung pats (Mann–Whitney *U = *1,276.50, *p < *0.001), but was less abundant than in dry yak‐dung pats (Mann–Whitney *U* = 86.00, *p < *0.001). Diptera were rare in crane fecal samples but relatively abundant in fresh yak‐dung pats (Mann–Whitney *U < *0.01, *p < *0.001) and sub‐dry yak‐dung pats (Mann–Whitney *U = *52.50, *p < *0.001), but there was no significant difference with dry yak‐dung pats (Mann–Whitney *U = *1,892.50, *p* = 0.106).

## 4 DISCUSSION

### 4.1 Foraging habitat selection and diet of the Black‐necked Crane

We found that cranes preferentially forage in habitats with heavy yak‐dung presence and lower, uniform vegetation, despite these habitats having lower overall arthropod abundance and Coleopteran abundance than habitats with taller, more heterogeneous vegetation (Figures 2 and 4; Supporting Information Figure S3). In contrast, previous studies have shown that insectivorous birds generally forage in habitats with low vegetation height but high height heterogeneity (Atkinson et al., 2005; Devereux et al., 2004; Evans et al., 2006; Vandenberghe et al., 2009), which is thought to represent a balance between the trade‐off of arthropod abundance and prey accessibility. The differences between our study and previous research can be reconciled when yak dung is considered. In addition, cranes are often the largest bird species in their environment, capable of breaking apart livestock dung with their bills, which likely creates differences in prey accessibility between cranes and smaller grassland insectivores. Although we found a positive relationship between mean sward height/heterogeneity and total abundance of invertebrate prey in the ecosystem (Figure 4a and Supporting Information Figure S3a), which is consistent with previous studies (e.g., Dennis et al., 2008; Morris & Plant, 1983; van Klink et al., 2014), Black‐necked Cranes are not foraging in these habitats. Instead, cranes are preferentially foraging in grazed habitats, which, as a consequence, have higher numbers of dry yak dung.

The results of our study emphasize the putative importance of Coleopteran prey and yak dung for Black‐necked Cranes. These results are suggestive that dung‐associated arthropods may help mitigate the impacts of reductions in total arthropod abundance and Coleopteran abundance that result from grazing (Figure 4). At our site, dung appears to be an important ecological factor that creates suitable microhabitats for certain arthropods to flourish, such as Coleopteran adults and larva, which are outnumbered by other arthropod orders that are active on the grassland surface but a significant part of the Black‐necked Crane diet. This study compliments other studies of birds and mammals, suggesting that large herbivore dung provides an important supply of invertebrate food items for higher‐level consumers, and should be considered when assessing the relationships between vegetation structure, arthropod abundance, and habitat selection in insectivorous birds (Benton et al., 2002; Evans et al., 2006; McCracken & Foster, 1994; Williams, Salter, & Jones, 2011).

### 4.2 The role of domestic yak dung on the grassland

The association that we observed between Black‐necked Cranes and domestic yak dung reveals an unrecognized dynamic on the Qinghai–Tibet Plateau grassland. The presence of dung may increase localized invertebrate abundance around dung pats, enriching ground invertebrates (Supporting Information Figure S4; Atkinson et al., 2004; Fuller & Gough, 1999). It has been previously suggested that dung may directly attract certain invertebrate species, particularly dung‐breeding species of Diptera and Coleoptera (Curry, 1987; Helden, Anderson, Sheridan, & Purvis, 2010), which may attract higher‐order insectivorous predators (Avilés, Sánchez, & Parejo, 2002; Davis & Vohs, 1993), like cranes. Although Coleopteran larvae were relatively rare on the surface of the grassland (as sampled by pitfall traps), they are abundant in the feces of the Black‐necked Crane (second only to Coleopteran adults, Figure 3b) and dominate dry dung pats. It should be noted that it is possible that we are even underestimating Coleopteran larva (as well as caterpillars and worms) in the crane diet because they have fewer hard parts that would be conserved through digestion.

In our study area, Black‐necked Cranes were often observed opening up or turning over dry dung pats, which has also been observed in other birds (Bignal & Ovenden, 1996; Meyer, 1990). This pecking behavior may help the degradation of dung and accelerate the nutrient and energy transfer through the grassland ecosystem. Dung‐associated arthropods and birds may thus influence the energy and material transfer through the grassland ecosystem. Our study further suggests that dung from large herbivorous mammals has an important ecological role in shaping grassland communities, providing new insight into the role that dung plays in habitat selection of a vulnerable and charismatic species. Insectivorous and omnivorous birds often rely on invertebrate prey items with high protein content while breeding and raising offspring (Kuang, Cangjue, Li, Yang, & Liu, 2010), and the foraging strategy of birds at this stage may be affected not only by the trade‐off between prey accessibility and prey abundance but also by the distribution of yak dung or other livestock dung on the grasslands.

## CONFLICT OF INTEREST

None declared.

## AUTHOR CONTRIBUTIONS

Wei Liu, Yongjie Wu, and Jianghong Ran designed the research; Wei Liu and Chenhao Zhao collected the field data; Wei Liu, Yongjie Wu, and Bin Wang analyzed the data and made the figures; and Wei Liu, Yongjie Wu, and Shane G. DuBay led the writing of the manuscript. Wei Liu and Yongjie Wu contributed equally to this work. All authors contributed intellectually to the discussion framework of the manuscript and gave final approval for publication.

## Data Availability

All data generated or analyzed during this study are included in this published article (and its supplementary information files).
