# Supplementary material for: Dung‐associated arthropods influence foraging ecology and habitat selection in Black‐necked Cranes (Grus nigricollis) on the Qinghai–Tibet Plateau
Source: Ecol Evol. 2019 Jan 28;9(4):2096–105. doi: 10.1002/ece3.4904 (PMC6392495; doi:10.1002/ece3.4904)

# Additional file 1

**Table S1** The difference in vegetation and arthropods among three sampling areas for testing the relationship between sward height and arthropod abundance.

**Table S2** The characteristic of foraging micro-habitats of Black-necked Crane in the four breeding stages.

**Table S3** The correlation matrix among means sward height, the total arthropod abundance, Coleopteran abundance and the number of yak-dung in the field.

**Fig. S1** The sketch map of the ten quadrats in each plot where we test the relationship between mean sward height and arthropod prey abundance and field sampling design. Each replicate plot (about 0.03 km^2^ in size) had ten 200 m^2^ quadrats that were surveyed (a) . The 200 m^2^ square (b) shows the size of a square used to test the relationship of mean sward height and prey abundance and to test the difference of vegetation structure between foraging and random square. The four 50 m^2^ squares in Fig. S1b (yellow, green, blue and red) were used to facilitate counting and examing yak dung in each foraging or random square. These four smaller squares help to avoid missing dung in the larger square. The 15 locations (1 m^2^) in Fig. S1b were used to measure sward height and sward height heterogeneity. We used the diagonal of the location (1 m^2^) as the transect. Surface-active invertebrates were sampled using a pitfall trap in five of the numbered locations (1, 2, 3, 4 and 5 in Fig. S1b).

**Fig. S2** Difference of arthropod abundance between fresh, sub-dry and dry yak-dung pats.

**Fig. S3** Relationships between sward height heterogeneity and (**a**) arthropod abundance, (**b**) number of dung pats and (**c**) foraging frequency in squares in four breeding stages. Fitted LOESS curves (50% of points fit) are shown for significant relationships in (**a**) and (**b**).

**Fig. S4** A summary of networks for energy and material transferring on the grassland ecosystem. Green arrows indicate positive relationships, red arrows indicates negative relationships. (**a**) A previous network of the potential relationship between invertebrates and livestock; (**b**) A new network showing the potential relationships between invertebrates and livestock, connected by the dung; (**c**) The main network of biomass when gazing is prohibited on the grassland; (**d**) The changed network of biomass when grazing is universal, in which dung and dung-associated insects and birds play important roles in maintaining biodiversity and ecosystem balance. (**c**) and (**d**) both indicate the livestock and invertebrates are competitors.

## **Table S1** The difference in vegetation and arthropods among three sampling areas for testing the relationship between sward height and arthropod abundance.

| Sampling areas | Number of samples | Mean sward height (cm) | Sward height heterogeneity | Number of Coleoptera adults | Number of all arthropod  individuals | Number of dung pats |
| --- | --- | --- | --- | --- | --- | --- |
| A | 120 | 15.99 ± 0.89 | 0.29 ± 0.02 | 34.53 ± 2.93 | 103.04 ± 5.58 | 1.59 ± 0.24 |
| B | 120 | 8.83 ± 0.46 | 0.1 ± 0.01 | 11.32 ± 1.55 | 59.63 ± 4.86 | 16.07 ± 0.68 |
| C | 120 | 5.64 ± 0.24 | 0.02 ± 0 | 6.04 ± 0.59 | 50.74 ± 2.69 | 27.82 ± 0.73 |

A: the first sampling areas which was winter pasture; B: the second sampling area which was rotational grazing pasture; C: the third sampling areas which was resettled habitat for tents and livestock (mean ± SE).

## **Table S2** The characteristic of foraging micro-habitats of Black-necked Crane in the four breeding stages.

| Breeding stage | Sample number | The proportion of the usage of different habitats | | |
| --- | --- | --- | --- | --- |
|  |  | Marsh | Marsh meadow | Meadow |
| i | 41 | 0 | 9 (22.0%) | 32 (78.0%) |
| ii | 40 | 1 (2.5 %) | 14 (35.0%) | 25 (62.5%) |
| iii | 36 | 4 (11.2%) | 13 (36.1%) | 19 (52.8%) |
| iv | 28 | 0 | 2 (7.1%) | 26 (92.8%) |
| Total | 145 | 5 (3.4%) | 38 (26.2%) | 102 (70.4%) |

All of 13 pairs in 145 observations. There were *Carex muliensis* community and *Carex muliensis*-*Cremanthodium stenactlninm* community in Marsh habitat, *Carex muliensis*-*Caltha scaposa* community in Marsh meadow habitat. There were *Kobresia tibetica*- *Kobresia capillifolia* community, *Kobresia tibetica*- *Trollius ranunculoides* community, *Kobresia capillifolia*- *Potentilla* anserine community, and *Elymus nutans*- weed community in Meadow habitat. i: Pre-incubation stage, ii: Incubation stage, iii: Post-fledging stage, iv: Full-fledged stage.

## **Table S3** The correlation matrix among means sward height, the total arthropod abundance, Coleopteran abundance and the number of yak-dung in the field.

|  | Mean sward height | The total arthropod abundance in the field | Coleopteran abundance in the field |
| --- | --- | --- | --- |
| Mean sward height | - |  |  |
| The total arthropod abundance in the field | Positively;  R^2^ = 0.67,  *P* < 0.001 |  |  |
| Coleopteran abundance in the field | Positively;  R^2^ = 0.75,  *P* < 0.001 | Positively;  R^2^ = 0.71,  *P* < 0.001 | - |
| The number of yak-dung | Negatively;  R^2^ = 0.21,  *P* < 0.001 | Negatively;  R^2^ = 0.11,  *P* < 0.001 | Negatively;  R^2^ = 0.20,  *P* < 0.001 |

## **Fig. S1**

## **
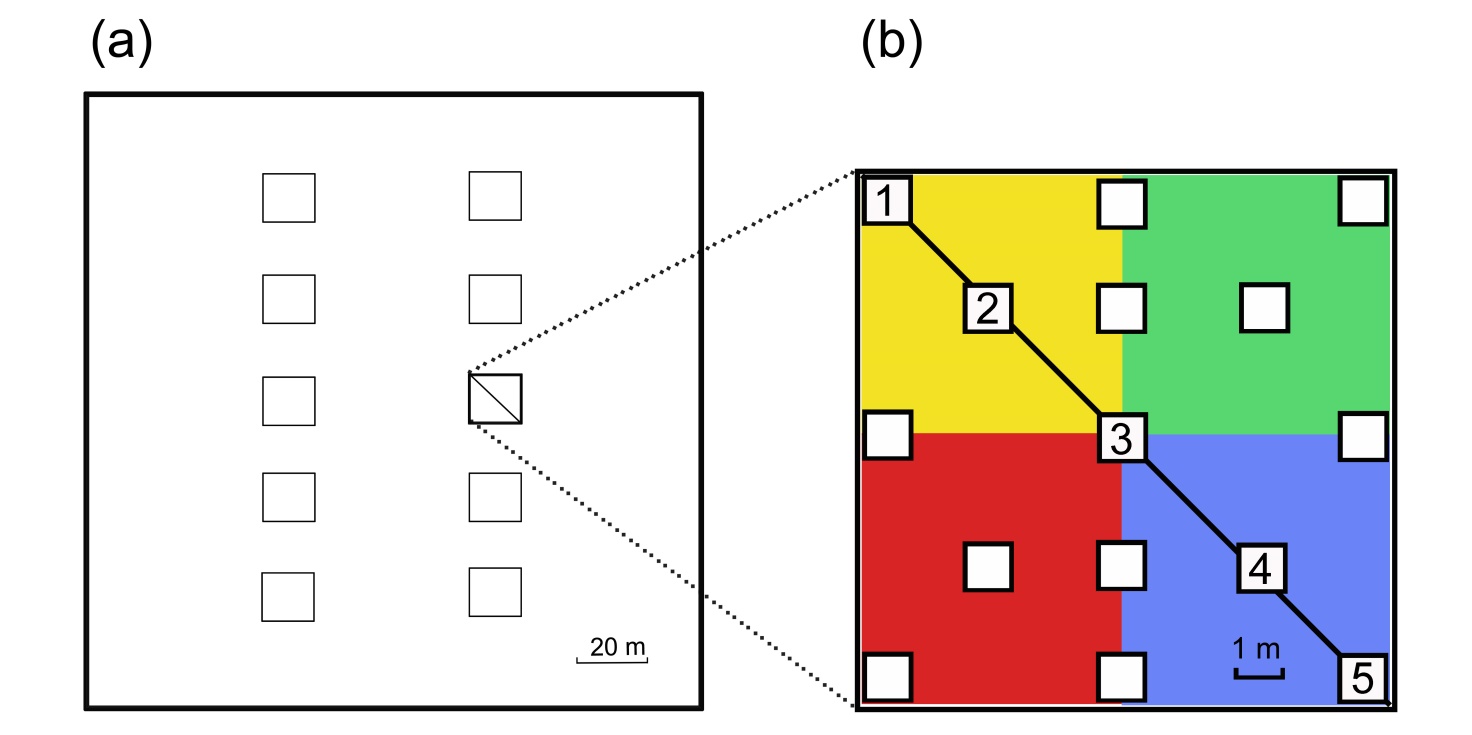
**

## **Fig. S2**


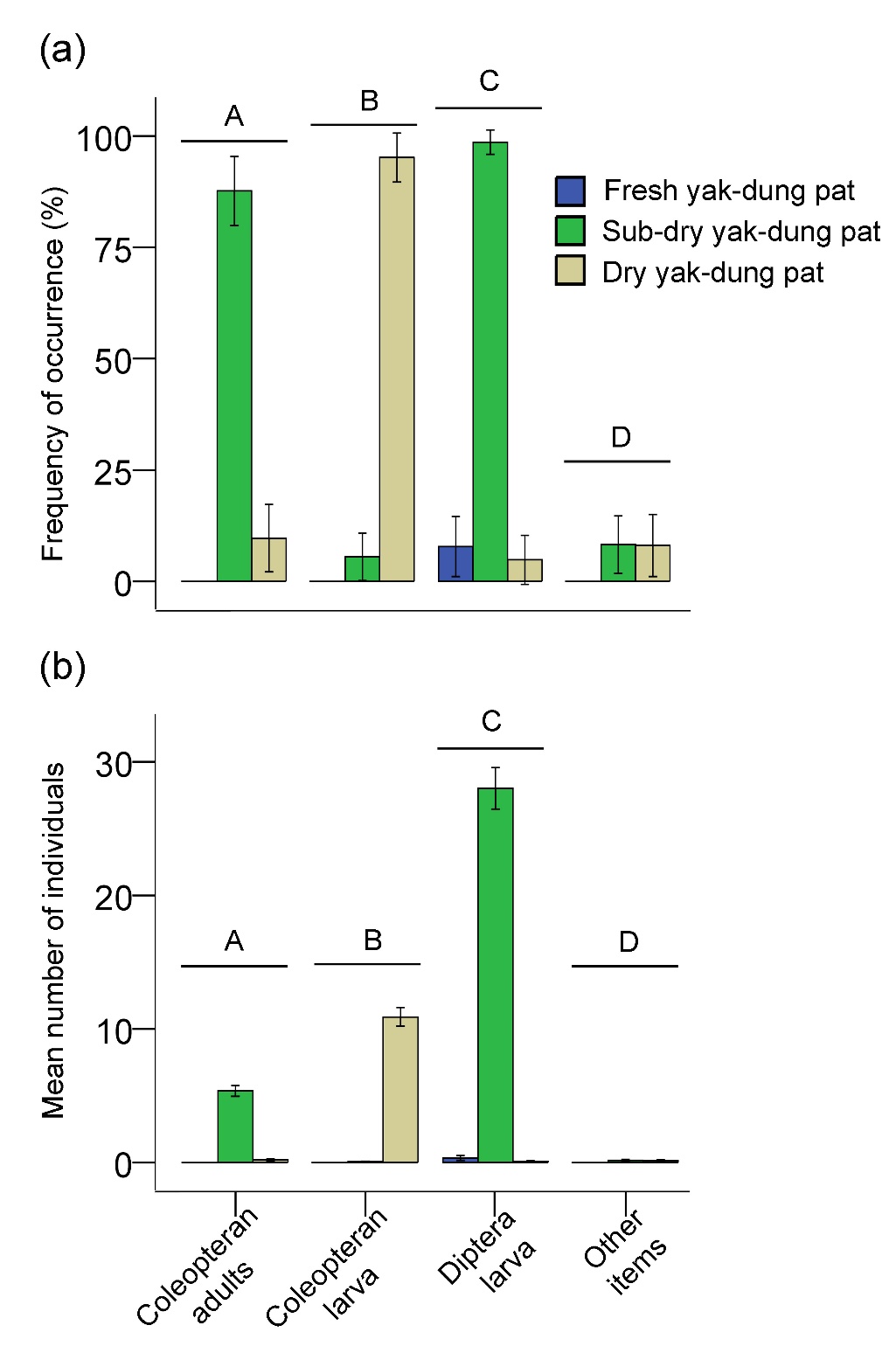


## **Fig. S3**


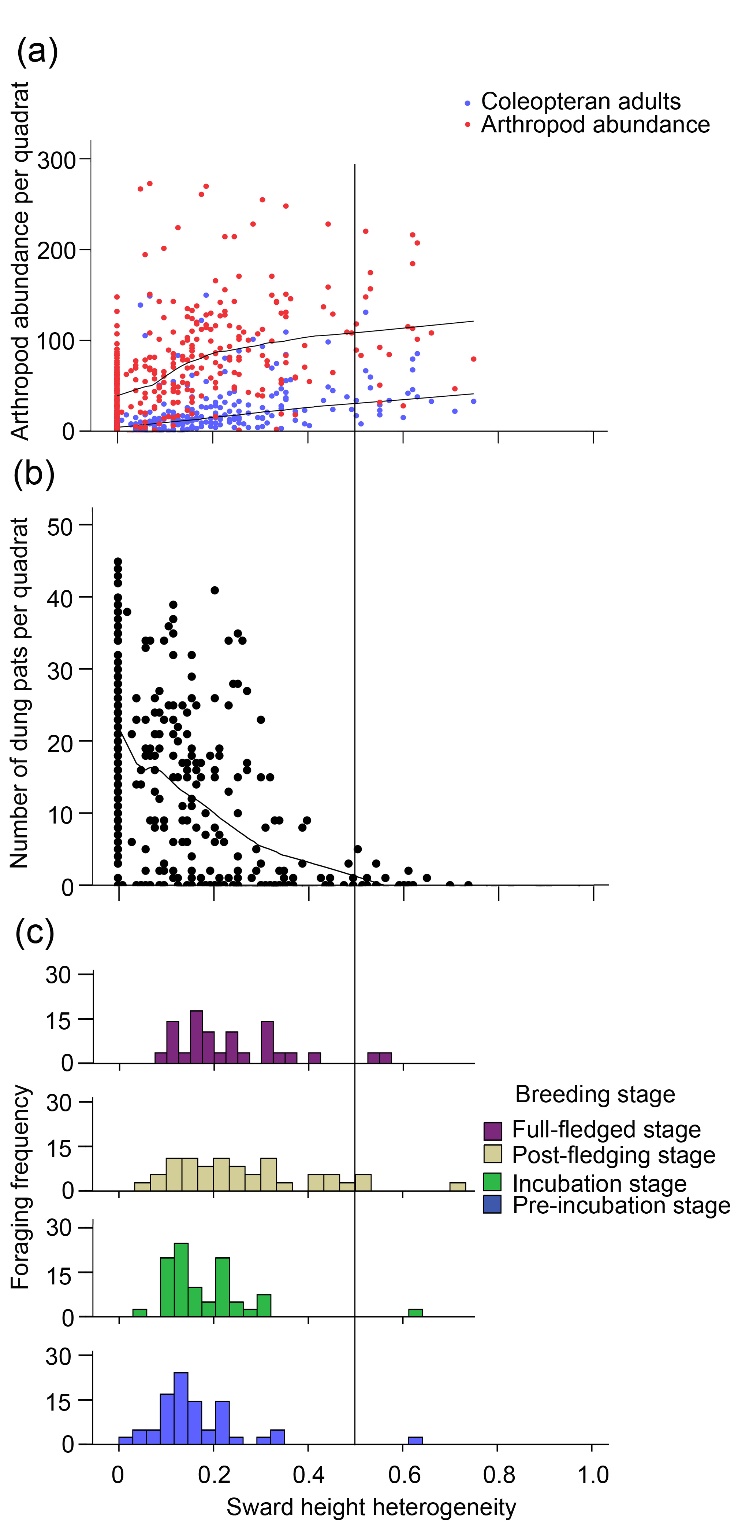


## **Fig. S4**


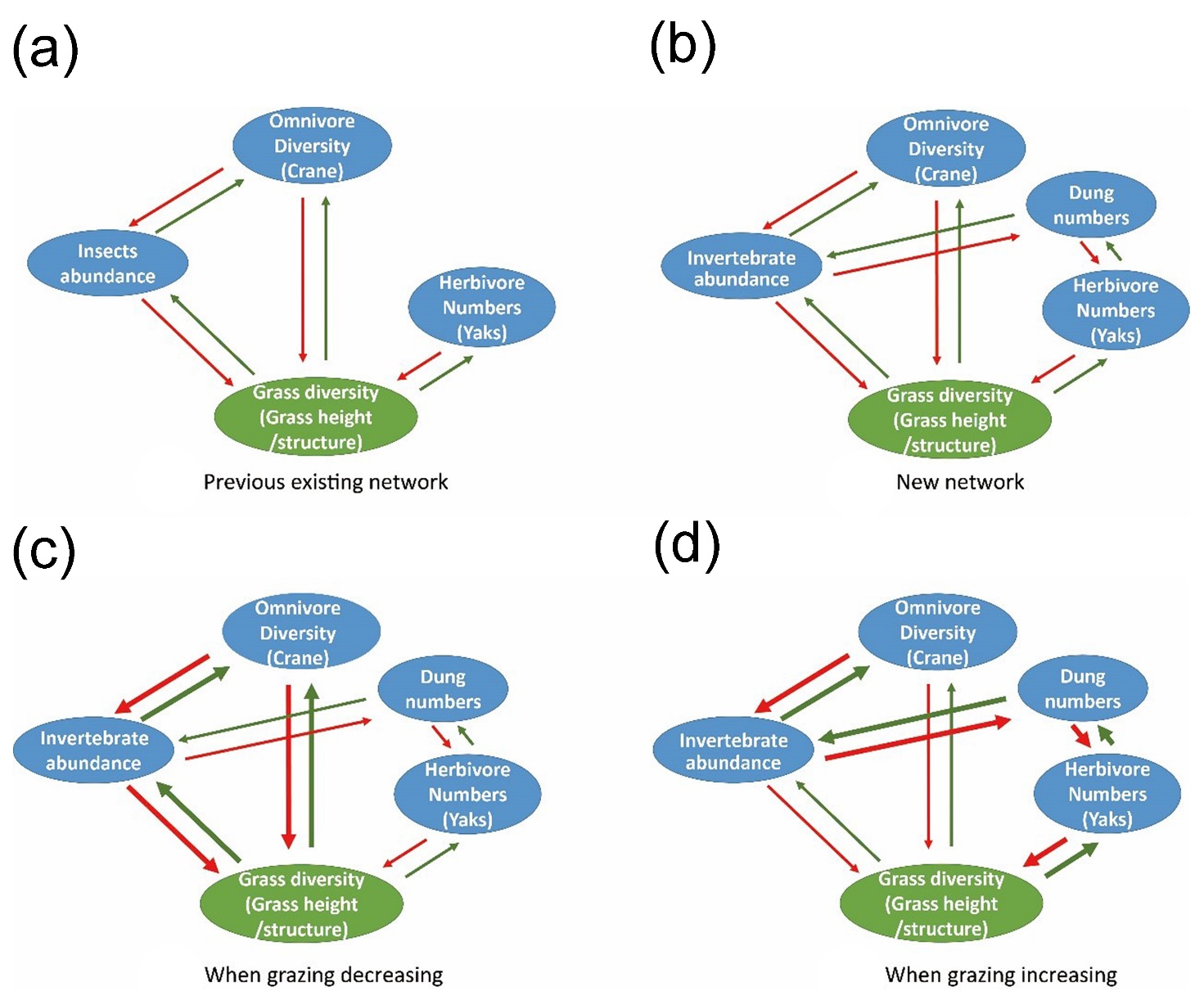

Supplement: Supplementary file 1 [file ECE3-9-2096-s001.docx]
